# Sentence-Level Effects of Literary Genre: Behavioral and Electrophysiological Evidence

**DOI:** 10.3389/fpsyg.2017.01887

**Published:** 2017-11-20

**Authors:** Stefan Blohm, Winfried Menninghaus, Matthias Schlesewsky

**Affiliations:** ^1^Department of Language and Literature, Max Planck Institute for Empirical Aesthetics, Frankfurt, Germany; ^2^Department of English and Linguistics, Johannes Gutenberg-Universität Mainz, Mainz, Germany; ^3^School of Psychology, Social Work and Social Policy, University of South Australia, Adelaide, SA, Australia

**Keywords:** ERP, N400, text type, genre, poetry, implicit prosody, schwa/zero alternation

## Abstract

The current study used event-related brain potentials (ERPs) and behavioral measures to examine effects of genre awareness on sentence processing and evaluation. We hypothesized that genre awareness modulates effects of genre-typical manipulations. We manipulated instructions between participants, either specifying a genre (poetry) or not (neutral). Sentences contained genre-typical variations of semantic congruency (congruent/incongruent) and morpho-phonological features (archaic/contemporary inflections). Offline ratings of meaningfulness (*n* = 64/group) showed higher average ratings for semantically incongruent sentences in the poetry vs. neutral condition. ERPs during sentence reading (*n* = 24/group; RSVP presentation at a fixed per-constituent rate; probe task) showed a left-lateralized N400-like effect for contemporary vs. archaic inflections. Semantic congruency elicited a bilateral posterior N400 effect for incongruent vs. congruent continuations followed by a centro-parietal positivity (P600). While N400 amplitudes were insensitive to the genre, the latency of the P600 was delayed by the poetry instruction. From these results, we conclude that during real-time sentence comprehension, readers are sensitive to subtle morphological manipulations and the implicit prosodic differences that accompany them. By contrast, genre awareness affects later stages of comprehension.

## Introduction

We use language to express ourselves, to refer to the external world, and to appeal to others (Bühler, 1934). Consequently, the selection and combination of speech sounds and meaningful elements serves to convey messages efficiently. Additional constraints on linguistic form derive from further communicative functions (Jakobson, 1960). For instance, in some types of *non-spontaneous* discourse – such as literature, rhetorical speeches, advertising, and other forms of propaganda – phonological form is consciously manipulated for a variety of purposes and effects (e.g., Wimsatt, 1944; McGlone and Tofighbakhsh, 2000; Menninghaus et al., 2014, 2015; Knoop et al., 2016).

Poetry is particularly interesting in this respect: as the writing process is not subject to the same practical constraints as daily communication, poets are free to create texts that satisfy a range of additional self-imposed formal constraints on the selection and combination of linguistic elements (e.g., Levin, 1962; Leech, 1969; Kintsch, 1998). Poetic meter, for instance, typically constrains the number of phonological elements (e.g., syllables or morae) that may occur in a line of verse, as well as the distribution of prominence features (accent, length, tone) across these elements (e.g., Fabb, 2015). Across centuries and literary traditions, poets have taken the liberty of deviating from the rules of a given language in order to meet self-imposed constraints, and of using newly coined, archaic, or otherwise deviant lexical and grammatical forms (Aristotle, 1911; Leech, 1969; Youmans, 1983; Sanni, 1993; Rice, 1997). The optional presence/absence of (historical) affixes, for example, provides creative options for altering the number of (unstressed) syllables. Some poetic traditions make extensive use of such options, particularly in languages that diachronically reduce complexity in their inflectional systems, e.g., English or German (Leech, 1969; Rice, 1997).

Semantics in poetry enjoys similar licenses. Poets assert that the language of poetry is “vitally metaphorical” (Shelley, 1840); some theoreticians claim that figurative speech and semantic polyvalence are *the* defining features of poetic texts (e.g., Thorne, 1988).

As a result, poetic discourse differs significantly from everyday verbal behavior (e.g., Wordsworth, 1802). However, sender and receiver typically convene in their conception of the type of discourse they are engaged in and, thus, both sides know how to act and what to expect (Beaugrande, 1978; van Dijk and Kintsch, 1983; Hanauer, 1998a,b; Kintsch, 1998). Genre awareness influences how we articulate a text (e.g., Byers, 1979; Barney, 1999), or how quickly we read it and what we recognize or recall of its surface structure and content (Zwaan, 1991, 1994; Hanauer, 1998b; Fischer et al., 2003; Tillmann and Dowling, 2007). The strongest evidence for genre-specific reading comes from studies that reported effects of genre attribution on the processing of identical texts (e.g., Zwaan, 1991, 1994; Schumacher and Avrutin, 2011).

But what is specific about poetry? Beaugrande (1978, p. 24) proposed that “readers have as part of the text-type frame the instructions to attend to sound recurrences in the assumption that these are not random.” Previous research on the processing of poetry has confirmed that sound recurrences influence how verbal stimuli are processed (Hoorn, 1996; Menninghaus et al., 2014; Obermeier et al., 2015; Chen et al., 2016; Vaughan-Evans et al., 2016) and evaluated (Van Peer, 1990; Obermeier et al., 2013; Menninghaus et al., 2014, 2016; Kraxenberger and Menninghaus, 2016). It can, however, not be taken for granted that these results indeed reflect *adapted* processing routines and *increased* attention to phonological structure due to genre awareness.

To address this question, the present study contrasted the processing of prosodic variation in identical sentence contexts with and without explicit genre attribution. We manipulated sentence prosody by means of a slightly archaic case suffix whose optional presence or absence constitutes one of German poetry’s routine licenses for balancing metrical structure. In addition, we manipulated the semantic congruency of the sentences to test whether the poetry mode of reading immediately affects the computation of meaning or whether semantic effects are to be found only at later stages of comprehension.

As previous studies on genre-specific reading have typically used global measures such as reading rate (Zwaan, 1991, 1994; Hanauer, 1998b), or investigated sentence-level categories such as newspaper headlines (Schumacher and Avrutin, 2011), it is still uncertain whether effects of literary genres are already detectable in the online processing of single sentences. Therefore, a main objective of the present study was to provide empirical evidence for genre-specific reading at the sentence level.

Note that while we are testing for genre-dependent adaptations that are *aprioristic*, we neither imply that adaptations necessarily precede processing (although categorization usually does), nor do we claim that they are exclusively genre-dependent. Rather, we assume that the schematic text type representations that experienced readers have acquired are associated with specific processing defaults (Zwaan, 1991, 1994; Hanauer, 1998b; Fischer et al., 2003; Schumacher and Avrutin, 2011), and that the textual variables of actual discourse may override these defaults (for semantic processing, see Nieuwland and Van Berkum, 2006). If this assumption is correct, and if we are correct in assuming that attention to sound recurrences and expectation of unusual semantic combinations are part of the poetic reading mode (Beaugrande, 1978; Thorne, 1988; Gibbs et al., 1991), then we should observe genre-dependent differences in the offline evaluation (Experiment 1) and online processing (Experiment 2) of verbal stimuli already at the level of the single sentence. If, on the other hand, genre-dependent adaptations are triggered by textual cues (e.g., graphic layout, prototypical structure, interlinear sound patterning), then the predicted effects should be independent of the instruction.

A secondary goal of Experiment 2 was to investigate potential effects of genre awareness on memory encoding by providing behavioral evidence from a probe recognition task: at the end of each trial participants were presented with a word, and indicated per button press whether or not it had occurred in the preceding sentence. Not only did this task ensure attentive reading, it also yielded information about the surface structure representation of the sentence after sentence wrap-up.

In previous research, enhanced memory of a text’s surface structure has been reported in delayed recognition and recall tasks for literary vs. non-literary texts (Zwaan, 1991, 1994; Hanauer, 1998b), and for poetry vs. prose (Hanauer, 1998a; Tillmann and Dowling, 2007). Tillmann and Dowling (2007, p. 636) argue that this effect is due to the systematic sound patterning that results in “the creation of richer and more precise memory traces,” and thus in more effective retrieval. While this interpretation is plausible, it is also possible that the poetic memory effect partially reflects differential memory encoding during processing of a given perceptual chunk. Thus, if we find a genre effect on recognition accuracy in the absence of the intricate sound patterning of a whole poem, this would constitute strong evidence for the idea that the poetic memory effect partially depends on an aprioristic modulation of the level of processing (e.g., Craik and Lockhart, 1972; Craik and Tulving, 1975).

## The Present Study

### Design and Materials

#### Design

These questions were addressed with a mixed design: instruction was manipulated between participants, but sentence prosody and semantic congruency were crossed within participants. Participants were assigned to one of two instruction groups: Instruction was either *neutral* with respect to text type (Neutral_INST_), or it identified the stimuli as verses, i.e., as single lines of *poetry* (Poetry_INST_). Sentences differed in terms of rhythmical regularity induced by alternative Dative^1^ case markers (Zero_DAT_ = irregular; Schwa_DAT_ = regular) and Semantic congruency (Congruent_SEM_ vs. Incongruent_SEM_). Note that both rhythm patterns occur naturally in everyday speech, but that only the Schwa_DAT_ pattern features strict rhythmical alternation (cf. **Table 1**).

**Table 1:** Example materials.

Instruction	Condition *(within participants)*	Example sentence
Neutral (*n* = 24) or Poetry (*n* = 24)	Schwa_DAT_/Congruent_SEM_	[In **die**sem **Be**tte]_PP_ [**schnarcht**]_verb_ [das **Mä**dchen]_NP_ [**laut**]_ADV_. lit: [In this_DAT_ bed_DAT_] [snores] [the_NOM_ girl_NOM_] [loudly] *In this bed, the girl snores loudly.*
	Schwa_DAT_/Incongruent_SEM_	[In **die**sem **Be**tte]_PP_ [**schnarcht**]_verb_ [das **La**ster]_NP_ [**laut**]_ADV_. lit: [[In this_DAT_ bed_DAT_] [snores] [the_NOM_ vice_NOM_] [loudly] *In this bed, vice snores loudly.*
	Zero_DAT_/Congruent_SEM_	[In **die**sem **Bett**]_PP_ [**schnarcht**]_verb_ [das **Mä**dchen]_NP_ [**laut**]_ADV_. lit: [In this_DAT_ bed_DAT_] [snores] [the_NOM_ girl_NOM_] [loudly] *In this bed, the girl snores loudly.*
	Zero_DAT_/Incongruent_SEM_	[In **die**sem **Bett**]_PP_ [**schnarcht**]_verb_ [das **Las**ter]_NP_ [**laut**]_ADV_. lit: [In this_DAT_ bed_DAT_] [snores] [the_NOM_ vice_NOM_] [loudly] *In this bed, vice snores loudly.*

*Example sentences and English translations for the four within-participant conditions; boldface indicates word stress; critical positions are underlined.*

#### Materials

We constructed 48 sets of sentences exemplified in **Table 1**. Sentences began with a prepositional phrase (PP) followed by a verb (V) and the subject noun phrase (NP). Four sentence variants per item set crossed Dative marking in the sentence-initial PP (Zero_DAT_ vs. Schwa_DAT_) and Semantic congruency of the subject NP (Congruent_SEM_ vs. Incongruent_SEM_); a full list of experimental stimuli is provided as Supplementary Material. For the EEG study, we inserted a postcritical adverbial (ADV) of one to four words after the critical NP position in order to avoid confounding effects of sentence wrap-up (Friederici and Frisch, 2000).

This configuration allowed us to use the initial PP as a prosodic prime that established an alternating rhythm pattern (trochaic or iambic)^2^. Thus, we modulated prosodic expectations for the critical verb in line with the Principle of Rhythmic Alternation, a preference for, and a tendency toward, the harmonious alternation of stressed and unstressed syllables (Jespersen, 1933; Selkirk, 1984; see e.g., Bohn et al., 2013; Henrich et al., 2014 for online evidence). This sentence structure further ensured that verb information was already available when the subject NP was encountered, which allowed us to manipulate semantic congruency by varying the semantic fit between verb and subject.

Consistent with the practice of poetic license in lyrical poetry, we used historical vs. contemporary case suffixes in sentence-initial constituents to manipulate word prosody without altering syntax or semantics. This morpho-phonological manipulation exploited a peculiarity of German grammar: the so-called *schwa/zero alternation* (Szczepaniak, 2010; Wiese and Speyer, 2015). Two dative case suffixes coexist in the inflectional paradigm of strong masculine and neuter nouns, one realized as a reduced vowel (Schwa_DAT_), the other without overt marking on the noun (Zero_DAT_). Synchronically, Zero_DAT_ marking constitutes the default form, being far more frequent and preferred in most registers of contemporary spoken and written German (Szczepaniak, 2010). However, the loss of the overt case suffix is a recent, ongoing development, so that the Schwa_DAT_ suffix has not been fully replaced. It is still used in formal and literary written registers, in non-casual speech, and in idiomatic expressions, and thus sufficiently familiar to any proficient speaker of German. Importantly, these word forms do not constitute violations; they are perceived as well-formed but stylistically marked, i.e., register-dependent, variants. According to a recent suggestion (Wiese and Speyer, 2015, p. 526), the variation “exists even in the standard language,” and “may exist in the same register of the language without any semantic or grammatical difference between the two forms.”

While functionally equivalent regarding grammar, these suffixes differ in terms of their effect on word prosodic structure: Zero_DAT_ marking does not affect the monosyllabic nouns we used, but Schwa_DAT_ marking adds a reduced syllable nucleus and results in trochaic word forms. This difference in word prosody motivates the seemingly free variation of these forms in German lyrical poetry, and it has been identified as one of the factors that co-determine their variation even in contemporary standard German (Wiese, 2000, 2016; Kentner, 2015; Wiese and Speyer, 2015). An example comparison of the two suffixes is provided in **Table 2**.

**Table 2:** Comparison of the two dative suffixes.

	Example	Stylistically marked	Number of syllables	Syllable structure	Stress on final syllable
Zero_DAT_	Tod (death_DAT_)	No	1	CVC	Yes
Schwa_DAT_	Tode (death_DAT_)	Yes	2	CV.CV	No

We used a variety of prepositions and modifiers to vary the form of sentence-initial PPs while keeping their basic constituent structure identical: [preposition – [modifier_DAT_ – noun_DAT_]*_NP_*]*_PP_*. All of the prepositions we used assign dative case to PP-internal noun phrases, and we carefully chose unambiguously case-marked modifiers (determiners, demonstratives or adjectives) to exclude confounding effects of case ambiguities.

Despite their length, we decided to present entire PPs at a time so they could serve as efficient prosodic primes. This presentation mode allows readers to immediately assign phrasal accent to the modified nouns in the PP, including monosyllables in the Zero_DAT_ conditions. Thus, we ensured that modified nouns were correctly stressed during silent articulation. We refrained from making predictions for the PP as we suspected that inference and interpretation might be complicated by two factors: (a) by the unusual three-word presentation mode that only allows extracting ERPs time-locked to the onset of the whole phrase (and not to the dative-marked noun alone), and (b) by potential temporal jitter, component overlap and component smearing that might result from inter-item differences in terms of prosodic complexity (3–6 syllables), as well as word classes (determiners vs. adjectives) and resulting conceptual complexity (one vs. two content words).

Verbs were identical across conditions. Critical verbs were monosyllabic and unambiguously marked for agreement with a third person singular subject. In line with the Principle of Rhythmic Alternation, dative case marking in the preceding constituent resulted in distinct prosodic preferences for the verb position. In Zero_DAT_ conditions, an accented monosyllabic noun precedes the verb and an unstressed syllable is preferred at the verb position. In Schwa_DAT_ conditions, a trochaic noun precedes the verb and results in a preference for a prominent syllable. Thus, monosyllabic verbs occurred in either a metrically prominent position (Schwa_DAT_) or in a non-prominent one (Zero_DAT_). As the polysyllabic word forms of these verbs (e.g., infinitives) are initially stressed, we hypothesized that lexical access would be facilitated if the monosyllabic forms occurred in a position expected to be prosodically prominent, i.e., in Schwa_DAT_ trials.

Subject NPs consisted of a determiner and a noun; determiners were marked for nominative case and indicated subjecthood. The event participant roles assigned by the verbs (e.g., *glance, weep, sit, laugh*) typically required a human/animate subject, i.e., an animate third-person singular subject NP was expected on the basis of the available verb information. The semantic congruency (Congruent_SEM_ vs. Incongruent_SEM_) of the NP was manipulated by varying the semantic fit between verbs and subjects: semantically incongruent sentences were constructed by replacing the nouns of the subject NP in the Congruent_SEM_ sentences while keeping determiners (i.e., morphosyntactic features) identical. The referents of the Incongruent_SEM_ nouns were less likely actors for the actions described (e.g., “*a robber/priest threatens with a dagger*”) and/or violated the animacy restrictions of the verb (e.g., “*a girl/vice sleeps in a bed*”). Inanimate nouns typically expressed abstract concepts (e.g., greed, innocence, vice), lending themselves more readily to a figurative reading than concrete objects (e.g., “*the screwdriver snores*”). The two noun sets were matched for lexical frequency^3^ and word length (both paired *t*s < 1.04; both *p*s > 0.27), but differed in terms of cloze probability (as assessed in an offline sentence completion task; *n* = 40): congruent nouns were more predictable than incongruent ones [paired *t*(47) = 3.84, *p* < 0.001]. Note, however, that the predictability of the critical nouns was very low in both the incongruent condition (range: 0.00–0.05; median = 0.00) and the congruent condition (range: 0.00–0.63; median: 0.03). In fact, more than two thirds of the congruent items had a cloze value of 0.05 or lower, which is typically considered very low contextual constraint. By comparison, a recent study that contrasted the processing of high and medium cloze probability words (Cermolacce et al., 2014) used item sets with mean cloze values of 0.95 and 0.46, respectively. In this respect, the current study differed from many previous ones: as predictability typically requires semantic congruency, the two variables are hard to dissociate experimentally and often confounded when effects of semantic incongruency are investigated.

## Experiment 1: Pen and Paper Meaningfulness Rating

The critical materials were pretested in a pen-and-paper rating study. Our aims were (1) to ascertain that our sentence variants indeed differed in terms of semantic congruency, and to test for potential effects of (2a) sentence prosody (=Dative) and (2b) genre awareness (=Instruction) on offline evaluation of meaningfulness. We tested the full set of critical stimuli. Note, however, that the sentences did not contain the postcritical adverbial we inserted for the EEG study based on methodological considerations. This ensured that observed differences in meaningfulness were most likely due to the semantic fit between verb and subject. Items were distributed over four lists so that each participant was presented with 12 sentences per condition and saw only one variant from each item set; four constrained randomizations were prepared for each of these lists. In addition to the resulting 48 critical sentences, each list contained 10 control items (5 highly implausible, 5 perfectly plausible) intended to represent the extreme ends of the scale, and enabling us to assess whether participants had understood the task.

### Hypotheses

Concerning aim (1), we expected a clear effect of semantic congruency with lower average meaningfulness ratings for Incongruent_SEM_ vs. Congruent_SEM_ sentences. Regarding aim (2a), we expected an effect of Dative on semantic evaluation only if phonological processing fluency is misattributed to perceived meaningfulness (McGlone and Tofighbakhsh, 2000). Our predictions regarding aim (2b) are based on a previous report of increased meaningfulness ratings for poetic vs. randomly generated metaphors (Gibbs et al., 1991). If these earlier findings reflect a *categorical* intentionality effect, i.e., an effect of intentional composition vs. random combination, we should not find genre-dependent differences. If, on the other hand, the search for additional significance forms part of the poetry schema, i.e., if assumed intentionality is *gradual*, we should likewise find increased meaningfulness ratings in the poetry instruction group, possibly restricted to semantically incongruent sentences prompting genre-specific interpretive strategies (Culler, 1975).

### Procedure and Data Analysis

A total of 128 members of the University of Frankfurt community were recruited on campus, all of them adult native speakers of German according to self-report (acquisition started no later than age 3). They were given a campus cafeteria coffee voucher as compensation for participation in the study, which took about 10–15 min. No further personal information was collected. Participants received one of two instructions, asking them to rate either “sentences” (Neutral_INST_) or “verses” (Poetry_INST_) on a 6-point rating scale ranging from 1 = *not meaningful^4^ at all* to 6 = *very meaningful*. English translations of the instruction texts are provided in **Table 3**.

**Table 3:** English translations of the instruction texts for the Neutral and the Poetry group in the rating study.

Neutral instruction	Poetry instruction
Thank you very much for your participation.	Thank you very much for your participation.
This study is part of a research project on **language**.	This study is part of a research project on **poetic language**.
In this questionnaire you are going to read a number of single **sentences**.	In this questionnaire you are going to read a number of single **verses^a^ selected from poems**.
Each of these expresses a situation or state of affairs.	Each of these expresses a situation or state of affairs.
Please read these **sentences** attentively and rate how meaningful they are using the scale next to the **sentence.** The scale ranges from “*not meaningful at all*” to “*very meaningful.*”	Please read these **verses** attentively and rate how meaningful they are using the scale next to the **verse**. The scale ranges from “*not meaningful at all*” to “*very meaningful.*”
Simply mark the box that, in your opinion, best locates the **sentence** on the scale.	Simply mark the box that, in your opinion, best locates the **verse** on the scale.
Please make sure to rate all of the **sentences**!	Please make sure to rate all of the **verses**!

*Boldface highlights differences between instructions.*

*^a^Unlike the English generic term ‘*verse*’, the German countable noun ‘*Vers*’ denotes a single line of poetry.*

We tested for main effects and interactions of Instruction, Dative, and Semantic congruency on ratings using linear mixed effects regression with crossed random effects for participants and items (Baayen et al., 2008). Reported *p*-value estimates are based on Satterthwaite’s approximation for degrees of freedom; *p*-values of multiple comparisons are FDR-corrected. Analysis was carried out in *R* (R Core Team, 2016) using the packages *lme4* (Bates et al., 2015) and *lmerTest* (Kuznetsova et al., 2016).

### Results and Discussion

Results of the meaningfulness rating are summarized in **Table 4**. Analysis confirmed that our critical word substitutions successfully manipulated semantic congruency. All of our Incongruent_SEM_ sentence versions were evaluated as less meaningful than their Congruent_SEM_ counterparts, with a mean difference of 1.94 (*SD* = 0.91) on the 6-point scale [*t*(5968.9) = -59.92, *p* < 0.001]. Analysis further revealed that participants assigned more meaning to the sentences if they believed to be reading poetic verses [*t*(127.5) = 3.08, *p* = 0.003].

**Table 4:** Results of the meaningfulness rating.

	Zero_DAT_		Schwa_DAT_	
	Congruent_SEM_	Incongruent_SEM_	Congruent_SEM_	Incongruent_SEM_
Neutral_INST_	5.25 (0.65)	3.20 (0.74)	5.23 (0.73)	3.21 (0.81)
Poetry_INST_	5.41 (0.51)	3.62 (0.70)	5.46 (0.49)	3.54 (0.75)

*Condition means (+*SD*s); 1 = not meaningful at all, 6 = very meaningful.*

**FIGURE 1:**
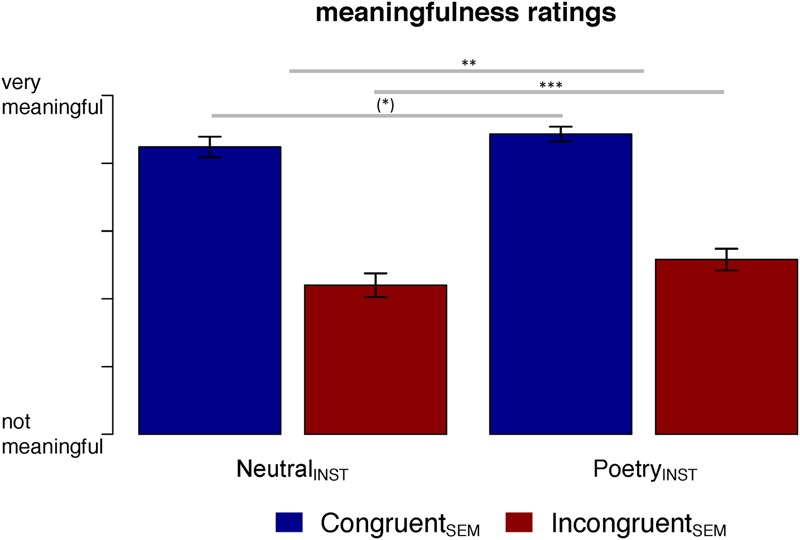
Mean ratings of meaningfulness for Incongruent_SEM_ and Congruent_SEM_ sentence variants by Instruction (Neutral_INST_ vs. Poetry_INST_; *n* = 64 each). Sentences were rated on a 6-point-scale ranging from “*not meaningful at all*” to “*very meaningful.*” Error bars indicate 95% confidence intervals; asterisks indicate statistical significance of planned comparisons: n.s., not significant; (^∗^) *p* < 0.08; ^∗^*p* < 0.05; ^∗∗^*p* < 0.01; ^∗∗∗^*p* < 0.001.

These simple effects were dissimilar within factor levels, as indicated by the significant interaction of Instruction and Semantic congruency [*F*(1,5968.9) = 8.35, *p* = 0.004]. Resolving the interaction by Semantic congruency, we found a trend toward a Poetry_INST_-induced increase in meaningfulness for Congruent_SEM_ sentences [*t*(160.3) = 1.96, *p* = 0.052]. In contrast, ratings for Incongruent_SEM_ sentences were reliably increased by the poetry instruction [*t*(160.3) = 3.86, *p* < 0.001], see **Figure 1**. Meaningfulness ratings were insensitive to the Dative manipulation [*F*(1,123.5) < 1].

Supplementing the findings of Gibbs et al. (1991), these results suggest that the poetry schema adds to the categorical intentionality effect: reading poetry entails the controlled search for additional significance beyond plain sense (e.g., Culler, 1975; Thorne, 1988), particularly if simple composition fails to yield a plausible/coherent representation of sentence meaning.

## Experiment 2: ERP Study

### Hypotheses

We expected to find modulations of the N400, an ERP component that has traditionally been linked to semantic processing (see Kutas and Federmeier, 2011 for a review). Based on previous ERP results on the processing of semantic incongruities (e.g., Federmeier and Kutas, 1999; Kutas and Hillyard, 1980), and based on the results of Experiment 1, we predicted increased N400 responses to Incongruent_SEM_ vs. Congruent_SEM_ subject noun phrases.

Crucially, amplitude and topography of the N400 – and to a lesser degree also its temporal characteristics – are sensitive to phonological processes that facilitate (or delay) lexical access, such as onset priming, rhyme priming, and prosodic priming. This has been demonstrated in auditory word, sentence, and text processing (e.g., Rugg, 1984a,b; Rugg and Barrett, 1987; Praamstra and Stegeman, 1993; Connolly and Phillips, 1994; Praamstra et al., 1994; Rothermich et al., 2012; Bohn et al., 2013; Henrich et al., 2014; Obermeier et al., 2015). For instance, Rothermich and colleagues reported modulations of N400 amplitudes depending on prosodic context, which they interpreted as evidence that the predictability of stress locations facilitates lexico-semantic processing. Importantly, N400-like prosodic negativities have been reported for silent reading as well (e.g., Luo and Zhou, 2010; Magne et al., 2010), in line with mounting evidence that *implicit prosody* (Fodor, 1998) co-determines the comprehension of written text (e.g., Bader, 1998; Steinhauer and Friederici, 2001; Stolterfoht et al., 2007; Breen and Clifton, 2011; Kentner, 2012; Kentner and Vasishth, 2016; Kriukova and Mani, 2016; see Breen, 2014 for a recent review).

Thus, we expected that strict rhythmical alternation (Schwa_DAT_) would allow predicting upcoming stress locations and thus facilitate lexico-semantic processing and result in reduced N400 responses (compared to Zero_DAT_) at the verb following the morpho-phonological manipulation.

Importantly, the N400 is also sensitive to task demands and varies as a function of the depth of processing (e.g., Rugg, 1984a; Bentin et al., 1993; Chwilla et al., 1995; West and Holcomb, 2000). Connecting this observation to Hanauer’s (1998b) hypothesis that the poetry reading mode modulates the level of processing, we further expected to observe distinct electrophysiological responses to morpho-phonological and semantic features in the two groups, if readers’ poetry schema entails adapted processing routines for either of these domains. More specifically, if poetry readers attend to sound recurrences in the assumption that these are not random, the poetry condition should exhibit more efficient use of prosodic cues and greater facilitation of rhythmic and/or lexico-semantic processing in favorable prosodic contexts (interaction effect: Instruction × Dative). This effect should be accompanied by a general reduction of N400 responses at both the verb and the subject NP (main effect: Instruction), if the genre-induced increase in attention to sound reduces the depth of semantic processing, parallel to the reduction of semantic effects due to task requirements (Bentin et al., 1993; Chwilla et al., 1995; Rothermich et al., 2012). If – as suggested by the results of Experiment 1 – unusual conceptual combinations are more expectable in poetic contexts, the system should downgrade contextual constraint on semantic access and lead to a reduced semantic N400 effect at the NP (interaction effect: Instruction × Semantic congruency). Alternatively, the interaction effect observed in Experiment 1 might be reflected in ERP differences in a late positive component, i.e., the P600. This component was long believed to be a marker of syntactic processing cost (e.g., Osterhout and Holcomb, 1992) but it also occurs in other contexts that are potentially relevant to the present study. First, the P600 is assumed to index the task-dependent evaluation of well-formedness (e.g., Bornkessel-Schlesewsky and Schlesewsky, 2008; Bohn et al., 2013). Second, P600 effects have been observed as part of a biphasic N400/P600 pattern in response to semantic incongruency and to animacy violations (e.g., Kutas and Hillyard, 1980; Szewczyk and Schriefers, 2011). Finally, the P600 is assumed to reflect a late pragmatic processing stage that follows semantic processing proper (as reflected in the N400), for instance in inferencing (Burkhardt, 2007), or in the computation of metaphorical meaning (e.g., Coulson and Van Petten, 2002; Weiland et al., 2014). More generally, the P600 and late positivities are believed to reflect controlled stimulus evaluation and the resolution of conflicts from prior processing streams (e.g., Bornkessel-Schlesewsky and Schlesewsky, 2008).

Our predictions concerning the probe task were as follows: if the processing system adapts attention allocation and memory encoding to the input category, i.e., the genre, then a memory effect should already be detectable in immediate recognition, leading to enhanced probe task performance for the poetry group (Poetry_INST_ > Neutral_INST_). If, on the other hand, we find no differences between the groups in terms of probe task performance (Poetry_INST_ = Neutral_INST_), this would support (but could not dissociate) the explanations that the memory effects observed in the delayed tasks of previous studies reflect slower reading and thus longer encoding (Hanauer, 1998b), or that they hinge on enhanced retrieval due to facilitating sound recurrences (Tillmann and Dowling, 2007). Our expectations concerning the Dative conditions depended on whether performance impairment due to infrequent morphology outweighs the benefits of facilitated prosodic processing (Zero_DAT_ > Schwa_DAT_) or vice versa (Schwa_DAT_ > Zero_DAT_). Furthermore, if the poetry schema licenses morphological deviations, we should find reduced impairment or enhanced facilitation of behavioral performance in this group (interaction: Instruction × Dative).

### Participants

Forty-eight monolingually raised native speakers of German (acquisition started no later than age 3) were recruited from the University of Mainz community and were paid 7 EUR per hour for participation in the experiment. All participants reported normal or corrected-to-normal vision and no known neurological or reading disorders, and all were right-handed as assessed with an abridged German version of the Edinburgh handedness inventory (Oldfield, 1971). Prior to the experiment, participants were informed about the experimental procedures and gave written consent. The data from four additional participants had to be excluded due to excessive body and/or eye movement artifacts. Participants were pseudo-randomly assigned to one of two instruction groups (Neutral_INST_ or Poetry_INST_). Care was taken that the groups matched in terms of the number of participants (*n* = 24 each) as well as sex (12 females per group) and age [Neutral_INST_: *mean* = 23.63, *SD* = 2.79; Poetry_INST_: *mean* = 24.83, *SD* = 3.57; two-sample *t*-test: *t*(46) < 1].

### Procedure

The 192 critical sentences resulting from the 48 item sets were distributed over two lists, each containing 96 critical sentences (2 chosen from each item set to total 24 sentences per condition) and 48 filler sentences^5^. Sentences that were taken from the same item set included one Congruent_SEM_ and one Incongruent_SEM_ continuation, with combinations of the two Dative variants rotated across items. Care was taken that at least 25 trials intervened between these pairs, and their order of occurrence was balanced across the two constrained randomizations we prepared for each list. All sentences conformed to the constituent structure described in Section “Materials.”

Participants received written instructions on a sheet of paper prior to the experiment; the experimenter ensured that instructions had been read and understood. Translations of the instruction texts are provided in **Table 5**. All experimental procedures were ethically approved by the Ethics Council of the Max Planck Society, and were undertaken with written informed consent of each participant.

**Table 5:** English translations of the instruction texts for the Neutral and the Poetry group in the ERP study.

Neutral instruction	Poetry instruction
Thank you very much for your participation in our **language** study.	Thank you very much for your participation in our **poetry** study.
You are going to read a number of single **sentences** on the screen.	You are going to read a number of single **verses selected from poems** on the screen.
Please read these **sentences** attentively.	Please read these **verses** attentively.
Before each **sentence** a little star (^∗^) indicates that the first part of the **sentence** is going to appear.	Before each **verse** a little star (^∗^) indicates that the first part of the **verse** is going to appear.
After reading a **sentence** you are going to be presented with a word.	After reading a **verse** you are going to be presented with a word.
Your task is to decide whether this word had occurred in the **sentence** or not.	Your task is to decide whether this word had occurred in the **verse** or not.

*Boldface highlights differences between instructions.*

Experimental sessions were conducted in a dimly lit and sound-attenuated room. Participants were seated comfortably at a distance of about 100 cm from a 17″ computer screen. Following a short practice session, the experiment consisted of four blocks of 36 sentences/verses each (∼8 min per block). Participants were encouraged to take short breaks (∼1–2 min) between blocks to relax and rest their eyes, as we had asked them to avoid movements and eye blinks during sentence presentation.

Sentences were presented constituent-wise in a 29-point font in the center of the display. Each trial began with the presentation of a fixation asterisk for 1000 ms, followed by a 200 ms inter-stimulus interval (ISI). Presentation rates were adapted to constituent size (e.g., Bornkessel et al., 2004; Schumacher and Hung, 2012): 450 ms for the PP (three words), 300 ms for the verb (one word) and 400 ms for the NP (two words), each followed by a 200 ms ISI. Presentation durations for the post-critical constituents also varied with length: one word = 300 ms (+200 ms ISI), two words = 400 ms (+200 ms ISI), three words = 450 ms (+200 ms ISI). Each trial ended with 500 ms of blank screen, after which participants performed a probe recognition task (cued by a question mark). In the probe task, participants were presented with a word, and indicated whether it had occurred in the sentence or not by pressing one of two pushbuttons on a hand-held gamepad; participants did not receive feedback on their performance during the experiment. The number of expected yes/no^6^ responses was balanced for each participant, as was the number of probes targeting each of the constituents. The assignment of left and right buttons to yes/no responses was counterbalanced across participants and within groups. The trial ended once a valid button press had been registered, or when the maximal response time (set at 5000 ms) had elapsed. After a further 1500 ms of blank screen the next trial started. A schematic depiction of this trial structure is provided in **Table 6**.

**Table 6:** Schematic trial structure (*bs* = blank screen) and presentation durations in ms.

Pre-stimulus		Sentence presentation								Probe task			
^∗^	*bs*	PP	*bs*	V	*bs*	NP	*bs*	ADV	*bs*	?	*bs*	probe	*bs*
1000	200	450	200	300	200	400	200	300–450	500	300	200	<5000	1500

### EEG Recording and Preprocessing

The EEG was recorded from 26 Ag/AgCl electrodes (ground: AFZ) fixed at the scalp by means of an elastic cap (Easycap GmbH, Herrsching, Germany). Four additional electrodes monitored the electro-oculogram (EOG) at the outer canthus of each eye (horizontal EOG) and above and below the participant’s right eye (vertical EOG). EEG and EOG channels were amplified by means of a BrainAmp amplifier (Brain Products, Gilching, Germany) and digitized with a sampling rate of 500 Hz. Recordings were referenced to the right mastoid but rereferenced to linked mastoids offline. Electrode impedance was kept below 5 kΩ during the experiment. We applied a 0.3–20 Hz band-pass filter to the raw EEG data offline in order to eliminate slow signal drifts and high frequency noise. The filter setting avoids potential stimulus-independent differences between compared conditions without performing a baseline correction which is susceptible to carrying over transient differences in the baseline period into the critical region (for a motivation of this approach, cf. Wolff et al., 2008; Schumacher and Hung, 2012; see also Widmann et al., 2015). We extracted ERP epochs from 200 ms before constituent onset to 1000 ms post onset before calculating single-trial averages for the latency bins of interest. Incorrectly answered and timed-out (>5000 ms) trials, as well as trials containing EEG or EOG artifacts (EOG rejection criterion: 40 μV; rejection rates: verb 1.2%, NP 1.9%), were excluded from further analysis (∼6% of the data).

### Data Analysis

For the statistical analyses of behavioral and ERP data, we tested for fixed main effects and interaction effects of Instruction, Dative and Semantic congruency using linear mixed-effects regression. For the ERP data of the verb position, where semantic information was not yet available, we only tested for fixed main effects and interaction effects of Instruction and Dative. Models contained crossed random effects for participants and items (Baayen et al., 2008). Random effect structure was determined using the forward-fitting algorithm implemented in the R package *LMERConvenienceFunctions* (Tremblay and Ransijn, 2015); the alpha level for the log likelihood ratio tests was set at *p* = 0.1. In order to facilitate interpretation and comparison, we report *F*- and *p*-value estimates based on the Satterthwaite approximation for degrees of freedom. Interactions were resolved hierarchically, controlling the false discovery rate at each level of analysis.

All analyses were carried out in *R* (R Core Team, 2016) using the packages *lme4* (Bates et al., 2015), *LMERConvenienceFunctions* (Tremblay and Ransijn, 2015), *lmerTest* (Kuznetsova et al., 2016) and *multcomp* (Hothorn et al., 2008).

#### Behavioral Data Analysis

##### Reaction times

We removed outliers exceeding 2.5 *SD*s from both the participant’s and the item’s mean before analyzing log-transformed reaction times of correctly answered trials. On the basis of these criteria, 7.6% of the original data points were excluded. The subsample was biased in the sense that, due to response accuracy, more Schwa_DAT_ trials were excluded than Zero_DAT_ trials [Chi-squared test for given probabilities: *χ^2^*(1) = 16.02, *p* < 0.001].

##### Accuracy

We tested for main effects and interaction effects of Instruction, Dative and Semantic congruency on response accuracy (correct/incorrect) using logistic regression.

#### ERP Data Analysis

ERP data analysis was carried out by fitting separate models for midline (FZ, FCZ, CZ, CPZ, PZ) and lateral electrodes (F3, F7, FC1, FC5, CP1, CP5, P3, P7, F4, F8, FC2, FC6, CP2, CP6, P4, P8). Outlying values exceeding ±20 microvolts were excluded from the analysis (∼1% data loss). For the lateral electrodes, four regions of interest were defined via combinations of the spatial factors Hemisphere (left/right) and Region (anterior/posterior). Contrast-coded spatial factors were included in the fixed effects term of the respective models, allowing for all interactions. Note that we do not report main effects and/or two-way interactions of spatial factors.

*Time window selection:* We predicted ERP differences in the N400 latency range (300–600 ms); the actual time windows for the analyses were chosen from this pre-determined latency range upon visual inspection of the grand average plots.

### Results

#### Behavioral Results

The behavioral performance suggests that participants generally processed the sentences/verses attentively: mean accuracy was 96.05%; mean response latency was 966 ms (1042 ms before outlier removal). A summary of the behavioral results is provided in **Table 7**.

**Table 7:** Behavioral results.

	Zero_DAT_			Schwa_DAT_		
		Congruent_SEM_	Incongruent_SEM_	Congruent_SEM_	Incongruent_SEM_
Neutral_INST_	*RT*	1003 (192)	976 (176)	971 (174)	1017 (194)
(*n* = 24)	*Error rate*	1.57 (2.41)	2.78 (2.92)	5.90 (5.20)	5.22 (5.26)
Poetry_INST_	*RT*	953 (224)	937 (192)	945 (202)	961 (197)
(*n* = 24)	*Error rate*	2.78 (3.82)	3.32 (3.26)	3.82 (3.23)	4.69 (5.11)

*Mean reaction times (RT) in ms and error rates per condition (*SD*s in parentheses).*

##### Reaction times

**FIGURE 2:**
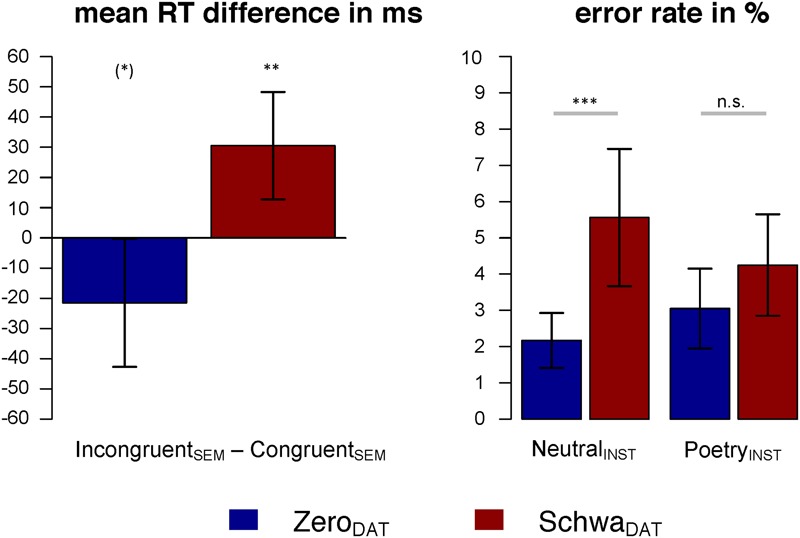
Behavioral results: Mean reaction time differences (Incongruent_SEM_ – Congruent_SEM_) in ms for the dative conditions (left panel), and mean error rates for the dative conditions by instruction (right panel). Error bars indicate 95% confidence intervals; asterisks indicate statistical significance of planned comparisons: n.s., not significant; (^∗^) *p* < 0.08; ^∗^*p* < 0.05; ^∗∗^*p* < 0.01; ^∗∗∗^*p* < 0.001.

Reaction times were on average 44 ms shorter for the Poetry_INST_ group (*M* = 949 ms, SEM = 36 ms) than for the Neutral_INST_ group (*M* = 993 ms, SEM = 41 ms), though this difference was not reliable [*F*(1,47.9) < 1]. Analysis further revealed that the effect of semantic congruency differed between levels of Dative [Dative × Semantics: *F*(1,4168.2) = 11.72, *p* < 0.001]. If sentences contained archaic Schwa_DAT_ case marking, reaction times were higher in Incongruent_SEM_ vs. Congruent_SEM_ trials [*t*(4168) = 3.00, *p* = 0.005]. Zero_DAT_ trials showed a trend toward a reaction time advantage for Incongruent_SEM_ vs. Congruent_SEM_ trials [*t*(4167) = -1.83, *p* = 0.067]; see **Figure 2**, left panel. Neither the main effect of Dative nor the interaction effect of Instruction and Dative approached significance (all *F*s < 1, all *p*s > 0.4).

##### Accuracy

There was a main effect of Dative on response accuracy: error rates were higher in the Schwa_DAT_ conditions than in the Zero_DAT_ conditions [*z* = -3.62, *p* < 0.001]. This effect differed between levels of Instruction [*z* = 2.06, *p* = 0.039]. Planned comparisons revealed that archaic Schwa_DAT_ nouns increased error rates in the Neutral_INST_ condition [*z* = -3.62, *p* < 0.001], but not in the Poetry_INST_ condition [*z* = -0.99, *p* = 0.324]; see **Figure 2**, right panel.

#### ERP Results

##### Verb

**FIGURE 3:**
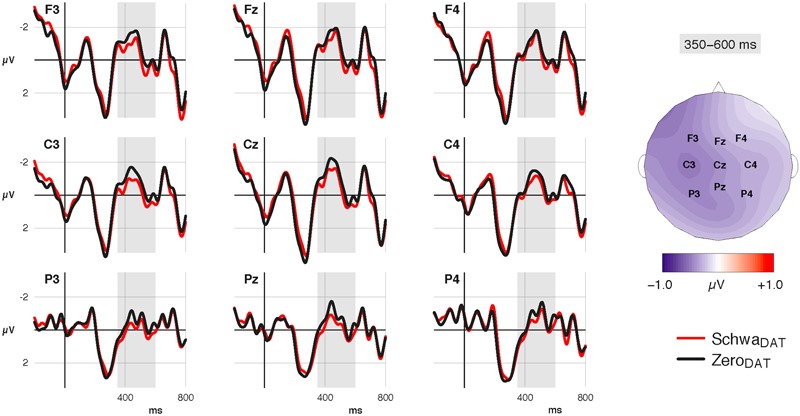
Grand-average ERP waveforms at the verb. Canonical Zero_DAT_ conditions show an increased negativity (350–600 ms) compared to non-canonical Schwa_DAT_ conditions.

*Time window 350–600 ms:* Verbs in the Zero_DAT_ conditions showed an increased negativity relative to verbs following a Schwa_DAT_-PP; the effect was widely distributed and most pronounced over left and central regions (see **Figure 3**). Statistical analysis confirmed a main effect of Dative marking at midline electrodes [*F*(1,45.971) = 12.22, *p* = 0.001, *t*(45.9) = -3.97] and lateral sites [*F*(1,46) = 15.77, *p* < 0.001, *t*(45.9) = -3.97], where the effect was further qualified by an interaction with Hemisphere [*F*(1,69917) = 7.88, *p* = 0.005]. Resolution verified that the effect was stronger over the left hemisphere [*t*(59.2) = –4.70, *p* < 0.001] than over the right hemisphere [*t*(59.2) = –2.76, *p* = 0.008]. None of the effects differed between instruction groups [lateral and midline: all *p*s > 0.25], nor were they affected by the repetition of the critical verbs [all *p*s > 0.36]^7^.

##### Subject NP

**FIGURE 4:**
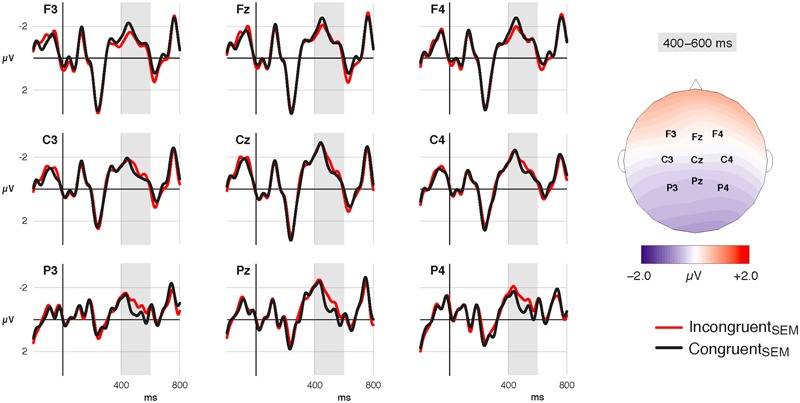
Grand-average ERP waveforms at the subject NP. Incongruent_SEM_ conditions show an increased bilateral posterior negativity (400–600 ms) compared to Congruent_SEM_ conditions.

**FIGURE 5:**
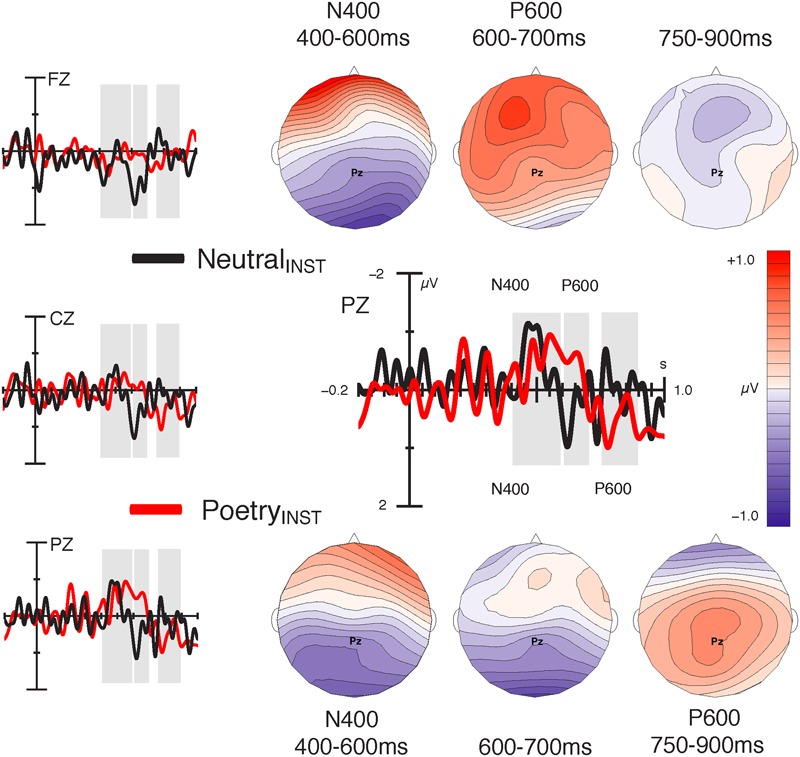
Instruction-wise comparison of semantic effects (Incongruent_SEM_–Congruent_SEM_) at the subject NP. Difference waves (left column) and difference maps illustrate semantic congruency effects for the Neutral_INST_ (black lines; upper row of maps) and for the Poetry_INST_ (red lines; lower row of maps) conditions. Both conditions show a posterior negativity (N400) between 400 and 600 ms. The late positive response (P600) occurs earlier in the Neutral_INST_ condition (600–700 ms) than in the Poetry_INST_ condition (750–900 ms).

Congruency conditions diverged around 400 ms after NP onset and showed a bilateral posterior negativity (N400) for incongruent vs. congruent NPs (see **Figure 4**). This effect was followed by late positive components (P600) whose onset latency differed between Instruction conditions (see **Figure 5**). In the neutral condition, incongruent NPs elicited a P600 between 600–700 ms after NP onset that was most pronounced over central and frontal sites. The poetry condition, in contrast, showed a sustained posterior negativity with an offset latency of ∼700 ms, followed by a centro-parietal positivity (P600) between 750 and 900 ms.

*Time window 400–600 ms:* Statistical analysis confirmed the visual impression: there was no semantic effect at midline electrodes [*F*(1,76.5) < 1, *p* = 0.440], but we observed an interaction of Semantic congruency and Region for lateral sites [*F*(1,68982) = 82.90, *p* < 0.001]. Resolving the interaction by Region verified a semantic negativity at posterior [*t*(48.1) = -3.44, *p* = 0.002], but no effect at anterior sites [*t*(48) = 1.60, *p* = 0.145].

*Time window 600–700 ms:* At midline electrodes, there was an interaction effect of Instruction and Semantic congruency [*F*(1,21523.3) = 24.12, *p* < 0.001]. Whereas the neutral condition showed more positive-going ERPs in response to incongruent vs. congruent NPs [*t*(73) = 3.37, *p* = 0.002], the poetry condition showed no difference [*t*(71.5) = -0.90, *p* = 0.371].

Laterally, this effect differed between Regions [*F*(1,68911.3) = 4.73, *p* = 0.030]. Absent in the posterior region [*F*(1,48) = 2.15, *p* = 0.149], an interaction effect of Instruction and Semantic congruency emerged over the anterior region [*F*(1,34360) = 28.85, *p* < 0.001], again reflecting more positive-going ERPs for incongruent vs. congruent NPs in the neutral condition [*t*(64.7) = 3.30, *p* = 0.003], and no difference in the poetry condition [*t*(63.6) = 0.30, *p* = 0.762].

*Time window 750–900 ms:* At midline sites, there was an interaction effect of Instruction and Semantic congruence [*F*(1, 21433.4) = 15.79, *p* < 0.001]. The neutral condition showed no semantic effect in this latency range [*t*(75.3) = -0.97, *p* = 0.335], but in the poetry condition ERPs were more positive-going in response to incongruent vs. congruent NPs [*t*(73.6) = 2.58, *p* = 0.024].

At lateral electrodes, we found an interaction effect of Instruction and Semantic congruency [*F*(1,69233.4) = 17.33, *p* < 0.001]. Resolution revealed a trend toward more-positive going ERPs in response to incongruent vs. congruent NPs in the poetry group [*t*(58.8) = 2.10, *p* = 0.080], and no difference between semantic conditions in the neutral group [*t*(59.5) = -0.53, *p* = 0.595]. Resolution of a significant interaction of Semantic congruency and Region [*F*(1,69147) = 8.87, *p* = 0.002] revealed no reliable effects [all uncorrected *p*s > 0.10].

## Discussion

Focusing on poetry and poetry-typical linguistic features, the present study aimed to investigate the effects of genre awareness on the processing and evaluation of isolated sentences. Differential instruction of participant groups allowed us to dissociate top–down-controlled genre effects from input-driven automatic processes.

In an offline rating study (Experiment 1), we validated our semantic manipulation and tested for effects of genre awareness on the perceived meaningfulness of the stimuli. Poetry categorization resulted in increased ratings of meaningfulness for semantically incongruent sentences.

In an ERP study (Experiment 2), we tested whether similar effects are detectable already in online processing. We expected (1) a prosodic N400-like effect at the verb due to facilitated lexico-semantic processing resulting from prosodic expectations built up during the processing of the sentence-initial constituent, and (2) a semantic N400 effect at the subject NP indexing the processing of incongruent meaning. We further hypothesized that (3a) the prosodic effect would be reinforced and that (3b) the semantic one would be attenuated by the poetry instruction. The results supported hypotheses (1) and (2) while hypotheses (3a) and (3b) were disconfirmed by the ERP data. The expected interaction of genre awareness and semantic congruency (3b) was reflected in the latency of the P600 response to incongruent vs. congruent NPs rather than in top–down modulations of N400 amplitudes. The interaction of genre awareness and morpho-phonological alternation (3a) was found in behavioral measures: rather than increasing facilitation, poetry instruction neutralized the adverse effect of stylistically marked case suffixes on response accuracy.

In the probe task, we investigated whether poetry categorization affects surface structure memory immediately after sentence wrap-up. Such an effect would provide strong evidence for the hypothesis that genre awareness modulates the level of processing and thus the resulting memory traces, which, in turn, could (partially) account for the enhanced surface structure recall and recognition reported for poetry vs. other text types. Results did not show the predicted general advantage for the poetry group and thus failed to support the level-of-processing account, at least in its strongest, aprioristic version. Regardless of the instruction, response latencies were delayed after processing sentences with combined morphological and semantic deviations. Response accuracy differed between instruction groups, reflecting that error rates increased with the presence of slightly archaic case markers when no genre had been specified, but not when sentences had been identified as lines of poetry. Most likely, this effect does not result from a modulation of the level of processing (which predicts a main effect of genre awareness), but merely reflects that stylistically marked case suffixes are more frequent and expectable in poetry than in most other registers. The results thus suggest that previously reported genre effects on surface structure memory are due to textual variables that were absent in the current study. First, the graphic layout of poetic texts seems to enhance the retrieval of surface structure representations of read poetry, possibly by providing spatial retrieval cues (Hanauer, 1998a). Second, the high degree of interlinear phonological patterning in poetic texts, e.g., line-final rhymes and recurrent meter, seems to increase memorability, as suggested by Tillmann and Dowling (2007). In this latter view, it is the systematic sound patterns that serve as effective retrieval cues in actual performance (Rubin, 1995), but also in experimental recall and recognition paradigms (Van Peer, 1990; Rubin et al., 1997; Hanauer, 1998b; Tillmann and Dowling, 2007; Lea et al., 2008). Note that this explanation is compatible with a level-of-processing account under the additional assumption that the depth of processing varies as a function of the amount of systematic sound patterning.

### Effects of Archaic Inflection: Behavioral Performance and Prosodic N400

As pointed out in the introduction, historical inflections provide poets with creative options for balancing the metrical structure of a line. To contemporary readers, however, they constitute morpho-phonological oddities, as indexed by the decline in subsequent probe task performance when no register/genre information had been provided. It seems that the sentence-initial presence of these infrequent word forms interferes with the behavioral task, possibly by introducing additional uncertainty concerning the exact form of the probe word. Importantly, this penalty diminished for participants in the poetry group. To us, this suggests that the poetry readers were better equipped to deal with poetry-typical, i.e., register-dependent, grammatical deviations while performing the task than were readers without previous text type information. Most likely, the genre instruction cued and activated the grammatical register associated with 18th/19th century lyrical poetry which is arguably the most canonical and prototypical representative of the German poetic tradition.

Notably, the morpho-phonological manipulation that impaired behavioral performance in one of the groups facilitated prosodic processing of the following word. In line with our predictions, we observed a reduced negativity in response to verbs following non-canonically case-marked NPs, resulting in a left-lateralized N400-like effect. It is unlikely that the observed effect is an instance of a semantic N400 that could be accounted for by semantic unification or contextual fit (e.g., Brown and Hagoort, 1993; Hagoort et al., 2009). First, the scalp distribution differed from the classic semantic N400 effect (Federmeier and Kutas, 1999). More importantly, our design allowed us to test the effect of the morpho-phonological manipulation on identical words embedded in semantically identical contexts within participants. But could this effect merely be a late positivity in response to the morphological archaism in the preceding constituent? We assume that this is not the case for two reasons. First, the late occurrence of the effect (∼1000–1250 ms after PP onset) makes such an interpretation questionable. Second, the case suffix does not constitute an ungrammaticality, i.e., it is not a violation but rather a register-dependent stylistic variant. Providing licensing register/genre information should thus modulate an effect that supposedly indexes the ill-formedness of a register-dependent alternation. Recall that the behavioral results seem to confirm that the poetry schema indeed licenses these historical inflections.

Therefore we assume that the observed negativity indeed indexes the relative ease of lexico-prosodic processing, i.e., of accessing and integrating a lexeme whose word prosodic template matches rhythm-based predictions (Rothermich et al., 2012; Henrich et al., 2014). This result aligns with earlier reports of phonological N400 effects in studies using auditorily and visually presented word pairs (Rugg, 1984a,b; Rugg and Barrett, 1987; Praamstra and Stegeman, 1993; Praamstra et al., 1994), single sentences (e.g., Luo and Zhou, 2010; Rothermich et al., 2012), and lyrical stanzas (Obermeier et al., 2015). If this interpretation is correct, our data extend the sentence-level effect of *implicit* prosody to comparatively subtle differences in morpho-phonological context, showing that the historical inflections frequently found in poetry successfully induce such differences.

The results do not support the idea that genre expectations affect prosodic processing in reading immediately and independent of textual variables. Previously reported effects of meter on poetry processing thus most likely reflect input-driven predictions based on phonological recurrence across lines. This interpretation is still compatible with the view that poetry reading affects top–down control of attention to phonological form. For the poetry group, our out-of-context lines thus correspond to the first line of a poem, where no metrical pattern has been established yet. While readers might expect a metrical pattern to be present, it is unclear, at this stage, which one it will turn out to be. Readers most likely extract the systematic sound patterning instantiated in a text incrementally when reading or hearing the first few lines or stanzas of a poem. This interpretation is in line with Smith’s (1968, p. 13) proposal that poetic structure is “an inference which we draw from the evidence of a series of events.” In this view, adaptations of prosodic processing are text-specific and recurrence-based rather than genre-specific and aprioristic.

Taken together, these results suggest that the same deviant morphological feature may impair and facilitate distinct stages of stimulus processing. This constitutes initial evidence from comprehension in support of the assumed trade-off between morphological deviation and prosodic regularity in poetic language (Rice, 1997). Our findings further suggest that genre awareness moderates the impairment of comprehension while leaving the facilitation intact.

### Effects of Semantic Congruency: Meaningfulness Rating, Semantic N400 and P600

Offline sentence evaluation confirmed the semantic congruency difference between sentence variants, and revealed a reliable interaction of instruction and semantics: genre categorization increased ratings of meaningfulness for incongruent sentences but not for congruent ones. These findings refine and extend the intention-based meaningfulness effect for metaphors (Gibbs et al., 1991) to non-metaphorical semantic incongruities. Rather than an intentional/non-intentional distinction, the present findings most likely reflect the genre-dependent aspect of the controlled search for hidden significance, and thus support a gradient conception of intentionality. These findings are in line with a two-step conception of genre-dependent semantic interpretation: upon failure to derive a coherent semantic representation by semantic composition (step one), poetry readers search for additional, hidden significance (step two).

Confirmed in ratings of meaningfulness, the semantic congruency difference also affected online sentence processing. In the ERPs, we observed a bilateral posterior N400 in response to semantically incongruent vs. congruent NPs. While the effect showed some characteristics that contrast with the “standard N400 effect,” i.e., a broadly distributed centro-parietal negativity between 250–550 ms (Kutas and Federmeier, 2011), its direction, its occurrence in the N400 latency range, as well as its familiar sensitivity to the semantic manipulation demonstrate clearly that we are dealing with an instance of a semantic N400.

As the N400 is highly sensitive to cloze probability (Kutas and Hillyard, 1984), we assume that the late onset of the effect most likely reflects the lack of lexical predictability in both congruency conditions. At the phrasal level, semantic congruency effects on the N400 differ from effects of predictability in terms of magnitude, latency and topography: while predictability differences elicit the familiar robust centro-parietal negativity, the N400 effects elicited by semantic incongruency are weaker and more variable in terms of scalp distribution (Lau et al., 2016). Furthermore, the restricted scalp distribution and the reduced magnitude of the present effect likely also reflect the large proportion of abstract nouns that we employed in the semantically incongruent conditions (e.g., *greed, grief, scorn, innocence, vice, curiosity*): abstract nouns are (a) known to elicit less pronounced N400 responses than concrete ones, typically with a more posterior distribution (Kounios and Holcomb, 1994; West and Holcomb, 2000), and (b) judged to be better event instigators than concrete ones and thus more likely to be realized as the grammatical subject – leading to less pronounced N400 responses if animacy expectations are not met (Frenzel et al., 2015). Finally, the sentence contexts in the present study did not exploit German native speakers’ high sensitivity to animacy in sentence interpretation (MacWhinney et al., 1984). German native speakers make use of an animacy heuristic in transitive sentences in which arguments compete for the event instigator role, particularly if deterministic morphological cues to interpretation (case/agreement) are unavailable (Frisch and Schlesewsky, 2001). In these contexts, animacy violations are especially aggravating, and lead to increased N400 amplitudes in response to assumed subjects/actors. In the present study, in contrast, the absence of competition for the actor role reduced reliance on the animacy heuristic, and thus potentially further attenuated the animacy-mismatch reflected in the N400.

While the semantic N400 effect was unaffected by genre categorization, the effect of semantic congruency on the P600 differed between genre conditions in terms of latency and scalp distribution, suggesting both temporal and functional differentiation in controlled conflict resolution. Indeed, it seems tempting to conclude that the P600 in the neutral group is a marker of well-formedness evaluation grounded in the default expectation of semantic congruency, and that the P600 in the poetry group reflects pragmatic processing triggered by the knowledge that superficially incongruent sentences may in fact be highly significant in poetic contexts. This explanation is in line with the idea that literary genres are conventionally associated with genre-specific interpretive strategies (Culler, 1975; Schauber and Spolsky, 1986) and it accounts parsimoniously for both the ERP pattern and the results of the meaningfulness rating. However, there are reasons to believe that the P600 effects and the processes that they reflect may, in fact, not be that different. First, they are remarkably similar in terms of their morphology, which suggests merely a delay of the same process. Second, the topography of the effect in the neutral group is unusual and potentially misleading since it quite likely reflects component overlap with the posterior N400. If so, the poetry condition would differ neither in terms of top–down control on semantic access nor necessarily in terms of the processes involved in controlled conflict resolution, but merely in terms of when conflict resolution is initiated. Thus, it is not entirely clear whether the late positivity effects merely index default vs. delayed processing, or whether they are indices of functionally distinct processing stages. In any case, the instruction-induced differences in late semantic evaluation match the effect of genre on the meaningfulness of incongruent sentences observed in offline evaluations, and thus corroborate the notion that later stages of comprehension are affected by the schematic genre representations that experienced readers have acquired.

## Conclusion

Our results disfavor the notion of aprioristic genre-specific adaptation of prosodically and semantically mediated lexical access if reading time is controlled for. It remains an open question, though, whether genre affects the predictability component of the N400. Regarding the semantic manipulation, our findings indicate that literary and non-literary real-time comprehension differ in the controlled evaluation of verbal stimuli although the exact nature of this difference needs to be addressed in future research. Regarding the morpho-phonological manipulation, they support the view that the subtle metrical differences induced by morphological licenses in poetry effectively modulate readers’ implicit prosodic expectations, and that genre awareness moderates the adverse effects of these poetic licenses.

## Author Contributions

SB, WM, and MS developed the hypotheses. SB and MS developed the study design. WM provided the funding. SB developed the materials, collected the data and performed the data analysis. SB, WM, and MS interpreted the data. SB drafted the manuscript, and MS and WM provided critical revisions. SB, WM, and MS approved the final version of the manuscript.

## Conflict of Interest Statement

The authors declare that the research was conducted in the absence of any commercial or financial relationships that could be construed as a potential conflict of interest.

## Footnotes

1 Dative case prototypically marks recipients in transfer-of-possession events, as in: ‘*Pat gave the book* ***to John*’**. A range of prepositions also assign dative case, e.g. those expressing locations: [*auf* [*dem*_DAT_ *Dach*_DAT_]*_NP_*]*_PP_* = [*on* [*the roof*]*_NP_*]*_PP_*.

2 Trochaic = stressed-unstressed; iambic = unstressed-stressed.

3 Lexical frequency was operationalized as the number of occurrences in *Wortschatz*, an online corpus of contemporary written German provided and maintained by the University of Leipzig, Germany (http://wortschatz.uni-leipzig.de).

4 We used the term ‘*sinnvoll’*, which is not fully equivalent to English ‘*meaningful’*: the German term conceptually covers both ‘to make sense’ and ‘full of significance or purpose’.

5 Fillers were identical to the critical materials in terms of syntactic structure. They also contained implausible (50%) and metrically regular (50%) sentences. Contrary to the critical materials, fillers showed a greater variety of semantic and metrical variation. For instance, in filler sentences, implausibility sometimes arose PP-internally from semantic mismatch between adjective and noun (instead of semantic fit between verb and subject). Metrical variation included ternary meters (e.g., dactyls) and polysyllabic verbs with different word stress patterns.

6 *No-*response probes were either phonologically (50%) or semantically (50%) related to the constituents targeted by the corresponding *yes*-response probes.

7 To assess whether the effects differed between the first and second encounter of an item, we conducted additional analyses, fitting models that contained main and interaction effects of Block (1st half/2nd half of the experiment). Results confirmed the reported data pattern, and did not reveal additional repetition-sensitive effects involving the variables of interest. However, we found a repetition effect at midline [main effect Block: *F*(1,21711.6) = 9.87, *p* = 0.002] and lateral sites [main effect Block: *F*(1,69958) = 29.35, *p* < 0.001]: ERPs were less negative-going in the second half of the experiment [midline: *t*(21711.6) = 3.14; lateral: *t*(69958.4) = 5.41].
