# Supplementary material for: Sentence-Level Effects of Literary Genre: Behavioral and Electrophysiological Evidence
Source: Front Psychol. 2017 Nov 20;8:1887. doi: 10.3389/fpsyg.2017.01887 (PMC5701934; doi:10.3389/fpsyg.2017.01887)
Supplement: Supplementary file 1 [file Table_1.pdf]

Supplementary Materials: Full list of critical sentences.

Dative conditions differed in terms of case marking in the sentence-initial PP (zero/-e).
Semantic conditions differed in terms of the subject NP's congruency.

ITEM	PP	V	NP_congruent	NP_incongruent	Post-critical continuation
1	Vom alten Turm(e)	**bläst**	**der Sturm**	**der Farn**	laut heulend
2	Vom grauen Berg(e)	**blickt**	**der Hirsch**	**der Wald**	hinab
3	Von dem Wind(e)	**bricht**	**der Zweig**	**der Bann**	entzwei
4	Auf jenem Feld(e)	**dörrt**	**der Mais**	**der Fleiß**	im Sonnenschein
5	Mit dem Dolch(e)	**droht**	**der Räuber**	**der Priester**	knurrend
6	Mit dem Kahn(e)	**fährt**	**der Fischer**	**der Gutmensch**	heimwärts
7	Vor jenem Krieg(e)	**flieht**	**das Volk**	**das Herz**	in Panik
8	Zu dem Fest(e)	**geht**	**der Tänzer**	**der Mörder**	unerkannt
9	Am wilden Bach(e)	**glänzt**	**der Tau**	**der Tag**	schon
10	In dem Sand(e)	**gräbt**	**die Krabbe**	**die Einfalt**	unaufhörlich
11	Mit hartem Stein(e)	**hält**	**die Brücke**	**die Sehnsucht**	immerfort
12	Auf dem Markt(e)	**herrscht**	**die Kundschaft**	**die Zwietracht**	eisern
13	Auf jenem Baum(e)	**hockt**	**der Affe**	**der Pfarrer**	träge
14	Vor dem Kreuz(e)	**kniet**	**die Nonne**	**die Sonne**	betend
15	Aus diesem Dorf(e)	**kommt**	**die Tante**	**die Ahnung**	her
16	Auf dem Dach(e)	**kräht**	**der Hahn**	**der Mond**	am Morgen
17	Nach dem Spiel(e)	**lacht**	**der Sieger**	**der Teufel**	schallend
18	Zu dem Tanz(e)	**lädt**	**der König**	**der Wohlstand**	ohne Zaudern

19	Am gleichen Fluss(e)	**lebt**	**die Nixe**	**die Mühe**	einsam
20	In dem Grab(e)	**liegt**	**der Sarg**	**der Traum**	verborgen
21	Im weisen Buch(e)	**liest**	**das Bübchen**	**das Kätzchen**	von der Poesie
22	An diesem Rock(e)	**näht**	**der Schneider**	**der Eifer**	fleißig
23	In dem Glas(e)	**perlt**	**der Sekt**	**der Senf**	verlockend
24	Bei frohem Werk(e)	**pfeift**	**der Meister**	**der Faulpelz**	trillernd
25	In tiefem Schlaf(e)	**ruht**	**die Frau**	**die Welt**	verwunschen
26	Bei edlem Wein(e)	**saß**	**der Zecher**	**der Becher**	singend
27	Vor der Tür(e)	**schläft**	**der Hund**	**der Baum**	am Abend
28	Im dunklen Wald(e)	**schleicht**	**der Fuchs**	**der Neid**	umher
29	Bei dem Mahl(e)	**schmaust**	**der Schlemmer**	**der Recke**	tagelang
30	In diesem Bett(e)	**schnarcht**	**das Mädchen**	**das Laster**	laut
31	In diesem Teich(e)	**schwimmt**	**die Ente**	**die Reue**	Tag für Tag
32	Bei diesem Klang(e)	**schwingt**	**die Luft**	**die Lust**	so herrlich
33	Mit frischem Mut(e)	**singt**	**der Chor**	**der Hund**	mit Inbrunst
34	Auf jenem Thron(e)	**sitzt**	**der Fürst**	**der Narr**	als Herr
35	An dem Strand(e)	**spielt**	**das Kind**	**das Glück**	am Meer
36	In dem Traum(e)	**spricht**	**der Schläfer**	**der Wahnsinn**	deutlich
37	Aus gutem Haus(e)	**stammt**	**das Fräulein**	**das Handwerk**	mit dem stolzen Namen
38	Vor deinem Bild(e)	**steht**	**die Tochter**	**die Neugier**	stundenlang
39	Nach dem Glück(e)	**strebt**	**die Jugend**	**die Tugend**	dauerhaft
40	In dem Saal(e)	**tanzt**	**die Braut**	**die Gier**	mit Anmut

41	Auf dem Meer(e)	**tobt**	**der Sturm**	**der Gott**	bei Nacht
42	Vom roten Blut(e)	**trinkt**	**die Zecke**	**die Trauer**	gierig
43	Auf meinem Haupt(e)	**wächst**	**das Haar**	**das Korn**	in Büscheln
44	Im fernen Land(e)	**weilt**	**die Gräfin**	**die Unschuld**	nimmermehr
45	Nach meinem Tod(e)	**weint**	**die Witwe**	**die Wirtin**	bitterlich
46	Auf diesem Schloss(e)	**wohnt**	**der Troll**	**der Groll**	vereinsamt
47	Mit blankem Gold(e)	**zahlt**	**der Graf**	**der Gram**	gewöhnlich
48	Nach großem Streit(e)	**zürnt**	**das Weib**	**das Wort**	noch Jahre
